# Evaluation of Bcl-2 Family Gene Expression and Caspase-3 Activity in Hippocampus STZ-Induced Diabetic Rats

**DOI:** 10.1155/2008/638467

**Published:** 2008-10-12

**Authors:** Iraj Jafari Anarkooli, Mojtaba Sankian, Shahriar Ahmadpour, Abdol-ReZa Varasteh, Hossein Haghir

**Affiliations:** ^1^Department of Anatomy and Neuroscience Research Centre, School of Medicine, Mashhad University of Medical Sciences, Mashhad, Iran; ^2^Department of Anatomy, School of Medicine, Zanjan University of Medical Sciences, Zanjan, Iran; ^3^Immunobiochemistry laboratory, Immunology Research Center, Bu-Ali Research Institute, Mashhad, Iran

## Abstract

We assessed the expression of Bcl-2 family members at both mRNA and protein levels as well as the Caspase-3 activity, in order to investigate the occurrence of apoptosis in hippocampus of STZ-induced diabetic rats. We selected twenty-four Wistar rats; half of them were made diabetic by intraperitoneal injection of a single 60 mg/kg dose of streptozotocin (STZ, IP), while the others received normal saline and served as controls. The expressions of Bcl-2, Bcl-x_L_, and Bax mRNA and proteins were measured using RT-PCR and western blotting, respectively. Caspases-3 activity was determined by using the Caspase-3/CPP32 Fluorometric Assay Kit. The result showed that mRNA and protein levels of Bcl-2 and Bcl-x_L_ were lower in hippocampus of diabetic group than that of the control group, whereas expressions of Bax in hippocampus of diabetic rats were higher than that of controls at both mRNA and protein levels (*P* < .01). Hyperglycemia was found to raise 6.9-fold hippocampal caspase-3 activity in diabetic group compared with control group (*P* < .001). Therefore, the induction of diabetes is associated with increased ratios of Bax/Bcl-2, Bax/Bcl-x_L_, and increased caspase-3 activity in hippocampus which shows that apoptosis is favored in hippocampal region.

## 1. INTRODUCTION

Diabetes mellitus is one of the most severe problems global, which is rising significantly. At the present, the WHO accounts 177 million patients with diabetes, while this number is expected to double in 2030 [1]. Diabetes mellitus is a heterogeneous metabolic disorder diagnosed by hyperglycemia resulting from impaired insulin secretion, resistance to insulin action, or both [2]. Hyperglycemia, which occurs under the diabetic condition, is often associated with complications, such as cardiovascular disease, renal failure, retinopathy and peripheral, and autonomic neuropathy [3, 4]. In recent years, it has been suggested that diabetes also affects the central nervous system [5, 6]. Within the central nervous system, the hippocampus is considered as a particular target for changes related to diabetes [7]. Hippocampus is a part of limbic system that serves as a critical integration center for cognitive functions such as learning and memory in the mammalian brain [8]. It has been reported that impairments in memory, learning, and cognition are more common in diabetic people than in nondiabetic ones. In type-1 diabetes mellitus, hippocampal neurons are extremely vulnerable [9–14]. Little is known about the mechanism by which hyperglycemia affects neurons [15].

Apoptosis could be proposed as a possible mechanism for hyperglycemia-induced hippocampal neuronal cell death. It has been revealed that is apoptosis implicated in several neurodegenerative disorders like Alzheimer's disease, Parkinson's disease, Huntington's disease, and amyotrophic lateral sclerosis [11, 16]. Apoptosis is a gene-regulated occurrence characterized by special morphological features, including condensation of chromatin, shrinkage of cell and nucleus, membrane blebbing, and DNA fragmentation [17]. Several factors are contributed in apoptosis, but the key elements are categorized in two main families of proteins including caspase enzymes and Bcl-2 family [18]. Caspase enzymes are working as a cascade and caspase 3 is the most important member of this family which plays an effective role in apoptosis of neurons of central nervous system [19]. Bcl-2 family is a set of cytoplasmic proteins that regulate apoptosis. The two main groups of this family, Bcl-2 and Bax proteins, are functionally opposed: Bcl-2 and Bcl-x_L_ act to inhibit apoptosis, whereas Bax counteracts this effect [19, 20].

In this study, we therefore assessed the expression of Bcl-2 family members at both mRNA and protein levels as well as the Caspase-3 activity, in order to investigate the occurrence of apoptosis in hippocampus of STZ-induced diabetic rats.

## 2. MATERIALS AND METHODS

We selected twenty-four male Wistar rats weighing (200–250 g) for this study. Animals were randomly divided into two groups (*n* = 12 in each group): the control (C) and diabetic (D) groups. They were kept in the animal house (Avicenna Research institute, Mashhad University of Medical Sciences, Mashhad, Iran) for one week for proper acclimatization before starting the experiment under controlled condition of illumination (12 hours light/12 hours darkness) and temperature 23 ± 2°C. They were housed under ideal laboratory conditions, maintained on standard pellet diet and water *ad libitum* throughout the experimental period. All procedures were in accordance with the Guide for the Care and Use of Laboratory Animals of Mashhad University of Medical Sciences, Iran.

Diabetes was induced in overnight fasted experimental group by a single intraperitoneal injection of STZ (Sigma, 60 mg/kg body weight) dissolved in normal saline, while the control group was injected with the normal saline only [21]. Three days after administration of STZ, the tail vein blood glucose level was measured in all animals. Blood glucose levels of 250 mg/dl and above were considered diabetic [22]. At the end of the eighth week, we measured weight and blood glucose level of rats. Then, animals were anesthetized with chloroform and the skull was open along the midline and the brain was removed and placed on an ice-cooled cutting board. The meninges were carefully removed and hippocampus was dissected from hemispheres, snap frozen in liquid nitrogen and stored at −70°C for extraction of DNA, RNA, and protein.

### 2.1. Isolation of total RNA and synthesis of cDNA

RNA was isolated from processed hippocampal tissue of rats using TriPure Isolation Reagent (Roche, Germany) according to the manufacturer instructions. Total RNA was reverse transcribed (RT) into cDNA with MBI RevertAid (Fermentase, Life Sciences, Lithuanian) according to the manufacturers instructions, and cDNA samples were stored at –20°C.

### 2.2. RT-PCR

A semiquantitative reverse transcriptase polymerase chain reaction (RT-PCR) was carried out to determine the levels of Bcl-2, Bcl-x_L_, and Bax mRNA expressions. The RT-PCR mixture (final volume of 20 *μ*L) contained 3 *μ*L of cDNA, 10 *μ*L of Qiagen Multiplex PCR Master Mix 2x (Qiagen, Germany) and 10 pmols of each complementary primer specific for Bcl-2, Bcl-x_L_, and Bax sequences as well as for GAPDH (Glyceraldehydes-3-phosphate dehydrogenase; GAPDH gene) sequence as an internal control (Table 1). The samples were denatured at 95°C for 15 minutes, and amplified using 40 cycles of 95°C for 30 seconds, 60°C for 80 seconds, and 72°C for 45 seconds for Bax gene and 5 cycles of 95°C for 30 seconds, 62°C for 60 seconds, and 72°C for 30 seconds, as well as 25 cycles of 95°C for 30 seconds, 64°C for 80 seconds and 72°C for 30 seconds for Bcl-2 and Bcl-x_L_ genes followed by a final elongation at 72°C for 3 minutes on a Corbett Research thermocycler (Sydney, Australia). The optimal numbers of cycles have been selected for amplification of all three genes experimentally so that amplifications were in the exponential range and have not reached a plateau. Five microliters of the final amplification product were run on a 3% ethidium-stained agarose gel and photographed. Quantification of the results was accomplished by measuring the optical density of the labeled bands, using the computerized imaging program Kodack-1D (USA). The values were normalized to GAPDH intensity levels.

## 3. WESTERN BLOT ANALYSIS

Tissues collected were homogenized with ice-cold homogenizing buffer (50 mM Tris-HCl, 150 mM NaCl, 1 mM EDTA, and 0.5 mM Triton X-100, PH 7.4) and protease inhibitor cocktail tablets (Roche, Germany). Proteins were measured with Bio-Rad protein assay method. Hippocampal protein lysates (50 *μ*g/well) were separated by SDS-PADE (12.5%) under reducing conditions and transferred to a polyvinylidene difluoride (PVDF) membrane (Millipore, USA). Membranes were treated with Attoglow western blot system kit, according to the manufacturer protocol (Biochain, USA). Briefly, blots were blocked with blocking buffer (5% not-fat dried milk in PBS). After blocking, blots were incubated with anti-Bcl-2 polyclonal antibody (1/500, v/v), anti-Bcl-x_L_ polyclonal antibody (1/1000, v/v), and anti-Bax polyclonal antibody (1/250, v/v) (Biovision, USA) for 20 hours at 4°C. Blots were washed for 4 times with 0.1% tween 20 in PBS and incubated with HRP conjugated-secondary antibody (1/5000, v/v, Biochain, USA) for 1 hour at room temperature. The Bcl-2, Bcl-x_L_ and Bax protein bands were visualized using enhanced chemiluminescnces (ECL) method (Bioimaging, system, syngene, UK).

## 4. DETECTION OF CASPASE-3 ACTIVITY

Caspase-3 activity was determined using the Caspase-3/CPP32 Fluorometric Assay Kit (K105) purchased from Biovision, Inc. (Mountain View, Calif., USA). For each assay, 50 *μ*g of tissue cell lysate was used. Samples were read in a fluorimeter equipped (Jasco, FP-6200, Japan) with a 400-nm excitation and a 505-nm emission filter. Fold-increasein Caspase-3 activity was determined by comparing fluorescence of 7-amino-4-trifluoromethyl coumarin in control and treated hippocampus with STZ.

## 5. STATISTICAL ANALYSES

All results are evaluated using the mean ± S.E.M. The results were analyzed with Mann-Whitney and paired *t*-tests. A probability level of *P* < .05 was considered significant.

## 6. RESULTS

Blood glucose concentrations of diabetic rats were more than 250 mg/dl 3 days after the administration of STZ. Blood glucose level at the end of the 8th week was significantly increased in diabetic group compared to nondiabetic (control) group (*P* < .001).

At first, both groups had similar body weights. Body weight was increased in the control group at the end of 8 weeks; however, the diabetic groups lost body weight compared to the control ones (*P* < .01) (Table 2).

### 6.1. Expression of Bax, Bcl-2 and Bcl-x_L_ genes at mRNA level

**Figure 1:**
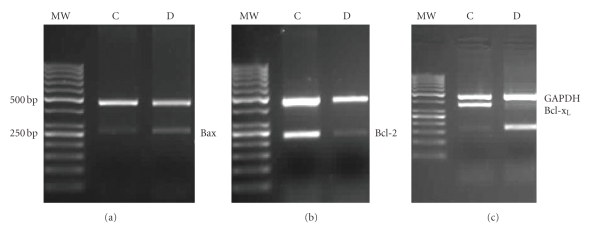
Semiquantative multiplex RT-PCR analysis of expressions of Bax, Bcl-2, and Bcl-x_L_ genes in hippocampus of control (C) and diabetic (D) groups of rat. (a) Amplification of the Bax gene (263 bp) compared with GAPDH gene (461 bp). (b) Amplification of the Bcl-2 gene (228 bp) compared with GAPDH (461 bp). (c) Amplification of the Bcl-x_L_ gene (357 bp) compared with GAPDH (461 bp).

**Figure 2:**
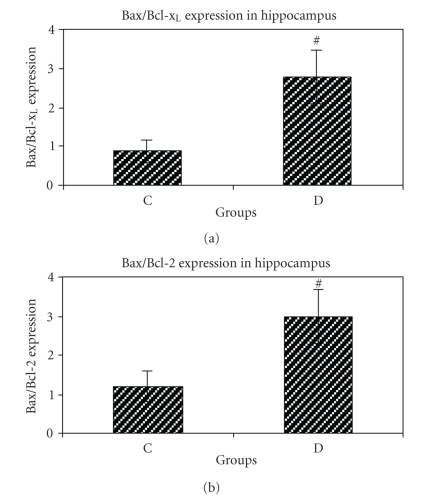
(a) Bax/Bcl-x_L_ ratio of control (C) and STZ-induced diabetic (D) groups of rat. Bar graph indicates the mean ± S.E.M.^#^ *P* < .01, compared to the control group (*n* = 4 each group). (b) Bax/Bcl-2 ratio of control (C) and STZ-induced diabetic (D) groups. Bar graph indicates the mean ± S.E.M.^#^ *P* < .05, compared to control group (*n* = 4 each group).

As shown in Figure 1, expression of Bax was increased in the hippocampus of STZ-induced diabetic group compared to that of control group, while the expressions of Bcl-2 and Bcl-x_L_ were reduced. Furthermore, the Bax/Bcl-2 and the Bax/Bcl-x_L_ ratios were increased in the hippocampus of STZ-induced diabetic group compared to control group (Figures 2(a) and 2(b)).

### 6.2. Expressions of Bax, Bcl-2, and Bcl-x_L_ proteins

**Figure 3:**
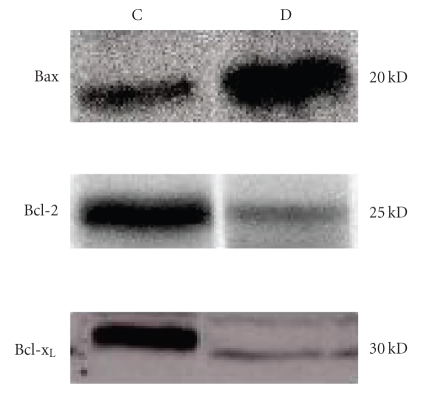
Western blot analysis to determine the expression of Bax, Bcl-2 and Bcl-x_L_ in extracts from hippocampus of control (C) and diabetic (D) groups of rat.

After eight weeks, the expression of proapoptotic Bax protein was enhanced in hippocampus of STZ-induced diabetic group compared to control group of rats. On the other hand, the expression of anti-apoptotic Bcl-2 and Bcl-x_L_ proteins were decreased in hippocampus of STZ-induced diabetic group compared to the control group of rats (Figure 3).

### 6.3. Evaluation of Caspase-3 activity in tissue extracts

**Figure 4:**
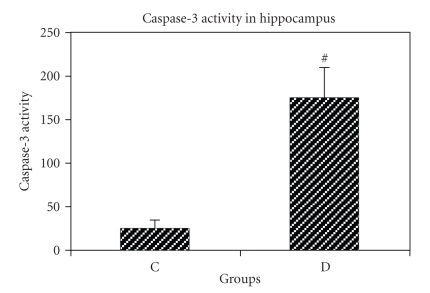
Caspase-3 activity in hippocampal tissues of control (C) and STZ-induced diabetic (D) rat groups. Bar graph indicates the mean ± S.E.M.^#^ *P* < .001, compared to the control group (*n* = 4 each group). Fold-increasein Caspase-3 activity was determined by comparing fluorescence of 7-amino-4-trifluoromethyl courmarin in control and treated hippocampus with STZ.

Caspase-3 activity was significantly higher in STZ-induced diabetic group compared to that of nondiabetic group (174.2 ± 36 versus 25 ± 10.25, *P* < .001). Hyperglycemia was found to increase 6.9-fold hippocampal tissue caspase-3 activity in diabetic group compared with nondiabetic group (Figure 4).

## 7. DISCUSSION

Despite of extensive investigations, the mechanism(s) activating apoptotic pathways in the diabetic brain have not been completely understood [23]. In the present study, we showed the effects of hyperglycemia on the expression of Bcl-2 family protein levels and Caspase-3 activity in hippocampus of STZ-induced diabetic rats.

Our results showed that after 8 weeks, Bax expression was considerably increased in hippocampus of STZ-induced diabetic rats at both mRNA and protein levels, while the expressions of Bcl-2 and Bcl-x_L_ were significantly reduced at both mRNA and protein levels. Furthermore, Bax/Bcl-2 and Bax/Bcl-x_L_ ratios, a main index of apoptotic cell death, were significantly increased; signifying hyperglycemia-induced apoptosis in hippocampus of STZ-induced diabetic rats could possibly be mediated by the mitochondrial pathway [24].

Other studies have demonstrated that diabetes induces apoptosis in central nervous system as well as in neuronal-related tissues, including peripheral nervous system, ganglions, and retina [11, 25, 26]. Several experimental models support that overexpression of Bax can induce apoptosis in cells both in vivo and in vitro [11, 27]. In addition, the apoptosis has been reported in neuroblast cell cultures treated with diabetic human sera [28]. Sharifi et al. showed that the excess concentration of glucose resulted in an increase in Bax expression protein and then induced apoptosis in PC12 cells [15]. Apoptosis and decrease in Bcl-2 expression were detected in primary neurons of dorsal root ganglion of spinal cord in diabetic rats [29, 30].

In this study, we were able to reveal the activation of proapoptotic proteins and suppression of antiapoptotic proteins in hippocampus of STZ-induced diabetic rats at both mRNA and protein levels. There are two classes of regulatory proteins in Bcl-2 family that substantiate opposite effects on apoptosis: the antiapoptotic members including Bcl-2 and Bcl-x_L_ which protect cells against some forms of apoptosis, and proapoptotic members including Bax, bak, and Bcl-x_S_ which progress programmed cell death. Apoptotic signals converge toward a common death pathway, for which caspases perform apoptosis and the Bcl-2 family proteins regulate it [31]. There is an absolute evidence that three main signals cause the release of apoptogenic mitochondrial mediators including a proapoptotic member of the Bcl-2 family, elevated level of intracellular calcium, and reactive oxygen species (ROS) or free radicals [32]. It has previously been revealed that both ROS and Nitric Oxide (NO) can change mitochondrial membrane potential by opening the mitochondrial permeability transition pores (mPTP), releasing cytochrome C and subsequent activation of caspases 9 and 3 and finally causing cell death. Members of the Bcl-2 family are known to be pro- and anti-apoptotic. The balance between pro- and anti-apoptotic signals from this family has a central role in the release of cytochrome C and its succeeding consequences [33].

Furthermore, we found that the activity of caspases-3, an effector of apoptosis that plays a key role to link the commitment and degradation phases [34], was significantly increased in hippocampus of STZ-induced diabetic rats compared to nondiabetic rats, which is consistent with previous studies [35–38].

Concurrent with our findings, Vincent et al. showed that hyperglycemia observed in diabetes mellitus was associated with oxidative stress-induced neuronal and Schwann cells death via increased caspase 3 activity [39].

The precise mechanism through which this occurs in hyperglycemia-induced apoptosis is not clear, but it has shown that hyperglycemia could elevate ROS in hippocampal neuronal cells resulted in elevation of intracellular calcium in neuronal cells [40]. Russell et al. showed excess mitochondrial ROS due to hyperglycemia depolarize mitochondrial membranes followed by decrease in ATP activity and increase in Caspases-3 and 9 leading to Caspase-dependent apoptosis [29]. Therefore, the induction of diabetes is associated with increased ratios of Bax/Bcl-2 and Bax/Bcl-x_L_ as well as increase of caspase-3 activity in hippocampus which shows that apoptosis is favored in hippocampal region. In our experiment we also alternative methods such as, silver staining for dark neurons, stereology, electron microscopy and DNA laddering to support the results that obtained with Bcl-2 family gene expression and caspase-3 activity (Unpublished).

## 8. CONCLUSION

Taking together, it may finally be concluded that the STZ-induced hyperglycemia causes hippocampal neuronal cell death, in which apoptosis plays an important role possibly by an increment in the Bax/Bcl-2 and Bax/Bcl-x_L_ ratio, as well as increasing caspase-3 activity. Diabetic-induced memory and cognition deficits may be partly due to a facilitation of apoptosis and this study provide further knowledge to mechanisms involved in STZ-induced apoptosis in hippocampus.

## Figures and Tables

**Table 1:** Primers used for RT-PCR method for Bcl-2, Bcl-x_L_, Bax, and GAPDH.

Genes	Primers	Product size
Bcl-2	F: 5′-CTG GTG GAC AAC ATC GCT CTG-3′	228 bp
	R: 5′-GGT CTG CTG ACC TCA CTT GTG-3′	
Bcl-x_L_	F: 5′-AGG CTG GCGATG AGT TTG AA-3′	357 bp
	R: 5′-TGA AAC GCT CCT GGC CTT TC-3′	
Bax	F: 5′-TTCATC CAGGAT CGA GCA GA-3′	263 bp
	R: 5′-GCA AAG TAG AAG GCA ACG-3′	
GAPDH	F: 5′-GGCCAAGAT CAT CCA TGA CAA CT-3′	462 bp
	R: 5′-ACC AGG ACA TGA GCT TGA CAA AGT-3′	

**Table 2:** Characteristic parameters of the control and STZ-diabetic rats. Values are shown as mean ± S.E.M. of 12 rats per group.

	Control	Diabetic
Initial body weight (g)	243.7 ± 9.2	247.0 ± 19.0
Final body weight (g)	306.0 ± 10.4	198.1 ± 21.0*
Initial blood glucose (mg/dl)	109.4 ± 5.6	106.9 ± 6.3
Final blood glucose (mg/dl)	111.2 ± 1.3	477.1 ± 28.4**

*P < .01 versus control.

**P < .001 versus control.
